# The *Pax *gene *eyegone *facilitates repression of eye development in *Tribolium*

**DOI:** 10.1186/2041-9139-2-8

**Published:** 2011-04-04

**Authors:** Nazanin ZarinKamar, Xiaoyun Yang, Riyue Bao, Frank Friedrich, Rolf Beutel, Markus Friedrich

**Affiliations:** 1 Department of Biological Sciences, Wayne State University, 5047 Gullen Mall, Detroit, MI 48202, USA; 2 Institut fur Spezielle Zoologie und Evolutionsbiologie, Friedrich Schiller Universitat Jena, Erbertstrasse 1, 07743 Jena, Germany; 3 Department of Anatomy and Cell Biology, Wayne State University, School of Medicine, 540 East Canfield Avenue, Detroit, MI 48201, USA

## Abstract

**Background:**

The *Pax *transcription factor gene *eyegone *(*eyg*) participates in many developmental processes in *Drosophila*, including the Notch signaling activated postembryonic growth of the eye primordium, global development of the adult head and the development of the antenna. In contrast to other *Pax *genes, the functional conservation of *eyg *in species other than *Drosophila *has not yet been explored.

**Results:**

We investigated the role of *eyg *during the postembryonic development of the red flour beetle *Tribolium castaneum*. Our results indicate conserved roles in antennal but not in eye development. Besides segmentation defects in the antenna, *Tribolium eyg *knockdown animals were characterized by eye enlargement due to the formation of surplus ommatidia at the central anterior edge of the compound eye. This effect resulted from the failure of the developing gena to locally repress retinal differentiation, which underlies the formation of the characteristic anterior notch in the *Tribolium *eye. Neither varying the induction time point of *eyg *knockdown nor knocking down components of the *Janus kinase*/*Signal Transducer and Activators of Transcription *signaling pathway in combination with *eyg *reduced eye size like in *Drosophila*.

**Conclusions:**

Taken together, expression and knockdown data suggest that *Tribolium eyg *serves as a competence factor that facilitates the repression of retinal differentiation in response to an unknown signal produced in the developing gena. At the comparative level, our findings reveal diverged roles of *eyg *associated with the evolution of different modes of postembryonic head development in endopterygote insects as well as diversified head morphologies in darkling beetles.

## Introduction

*Pax *transcription factor family genes constitute an important part of the genetic toolkit that controls the development of the metazoan body plan including the visual system [1-3]. Four *Pax *genes have thus far been found to be important for *Drosophila *eye development: The tandem duplicated *Pax6 *orthologs *eyeless (ey) *and *twin of eyeless (toy)*, which are critical regulators of early primordium specification and proliferation [4,5], the *Pax2/5/8 *paralog *shaven *(*sv*), which functions as the cone and pigment cell selector gene during retinal differentiation [6], and the *Pax *transcription factor gene *eyegone *(*eyg*), which has been discovered due to its essential requirement for retinal primordium growth [7,8]. *eyg *also has a tandem duplicated sister paralog in *Drosophila *named *twin of eyegone *(*toe*), which, however, plays only a subtle role in the *Drosophila *eye imaginal disc despite a spatially identical expression pattern [9].

While the deep functional conservation of *sv*, *ey *and *toy *in eye development has been extensively studied [10], it is unknown whether the eye growth activating function of *Drosophila eyg *has similarly deep evolutionary roots. In part, because of the lack of *eyg *orthologs in vertebrates, it has been hypothesized that *eyg *represents a functional homolog of the retinal primordium proliferation specific vertebrate *Pax6 *isoform 5a [11-13]. Recent analyses of *Pax *gene family evolution, however, uncovered *eyg *orthologs in hemichordates but not in lancelet fish, revealing that the apparent loss of *eyg *during early chordate evolution is not correlated with the emergence of *Pax6(5a) *in vertebrates in a manner that is consistent with compensatory replacement [14-16].

The *Drosophila eyg *and *toe *genes possess a paired domain (PD) and a homeodomain (HD) but lack an octapeptide domain, which is characteristic of select *Pax *gene subfamilies [12,17]. While the HD of *eyg *and *toe *falls into the paired class of HDs, their PD is unique. Canonical PDs have a tripartite structure defined by three DNA-contacting subdomains: the N-terminal PAI domain, the C-terminal RED domain and the central linker region [18-21]. In *Drosophila eyg *and *toe*, the PAI domain is N-terminally truncated, while the linker region is largely conserved and the RED domain completely intact [12]. The binding specificity of the *eyg *PD was proposed to resemble *Pax6(5a)*, which is believed to bind DNA only through the RED domain due to the introduction of an additional coding exon into the PAI domain [12]. However, recent sequence conservation analyses generated preliminary evidence that the presumptive PAI domains of *Pax6(5a) *and insect *eyg *retained DNA binding function [14].

**Figure 1:**
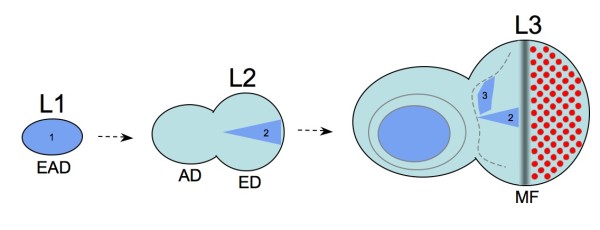
**Expression patterns and developmental functions of *eyg *in the *Drosophila *eye-antennal imaginal disc**. Blue areas describe approximate expression domains of eyg. The three separately controlled and functional expression domains are numbered. The homogenous expression domain 1 in the first instar eye-antennal disc as well as the more confined expression domain 3 in a dorsal area of the third instar eye disc are essential for global head development. Expression domain 3 has also been found to be required for the development of specific lateral head bristles [25]. Expression domain 2 in the second larval instar eye disc is induced by N-signaling as part of the gene regulatory network that drives essential proliferation of the eye primordium in the posterior eye disc. Expression domain 2 ceases in front of the anteriorly progressing morphogenetic furrow (MF). Data from references 22 and 25. AD = antennal disc, ED = eye disc, EAD = eye-antennal imaginal disc, L1 = first larval instar, L2 = second larval instar, L3 = third larval instar. Dorsal up and posterior to the right.

The genetic analysis of *eyg *in *Drosophila *pinpointed two *eyg*-dependent processes, which are essential for the development of the adult eye in this species. These functions are associated with three discrete expression patterns in the developing eye-antennal disc (Figure 1). The earliest expression of *eyg *in precursor cells of the adult eye is initiated in the nascent eye-antennal imaginal disc during embryonic development [22]. At this point, *eyg *belongs to a number of developmental transcription factors, which are expressed throughout the entire eye-antennal imaginal disc [22]. This global expression of *eyg *discontinues during the second larval instar and is followed by renewed induction of transcription along the dorsoventral midline of the second instar eye imaginal disc. This expression is, most likely indirectly [22], activated in response to localized Notch (N) signaling [7,8]. Enhancer-specific rescue experiments have shown that the midline expression domain is specifically required for the development of the retina [22]. The combined evidence from several studies suggests that this requirement is associated with two mechanisms. For one, *eyg *activates the expression of the ligand gene *unpaired *(*upd*), which results in activation of the *Janus kinase*/*Signal Transducer and Activators of Transcription (Jak/STAT*) signaling pathway, which then stimulates tissue proliferation in the eye disc [7]. In parallel, *eyg *initiated *Jak/STAT *signaling enforces the repression of *wingless *(*wg*) transcription in the posterior eye disc [17,23,24]. These data lead to a model in which *eyg *is part of an N-induced genetic cascade that potentiates the essential proliferation of retinal precursor tissue in the eye disc and generates competence for the induction of eye development by repressing the retinal differentiation antagonist *wg*.

During the third larval instar, a new discrete *eyg *expression domain is initiated in a broad dorsal area in front of the morphogenetic furrow [22], where *eyg *expression is necessary for normal development of specific bristles [25]. At the same time, the midline expression domain is successively repressed by the progressing morphogenetic furrow to the effect that *eyg *is not expressed in the early differentiating retina [22]. Interestingly, the expression of *eyg *anterior to the morphogenetic furrow does not seem to be specifically required for eye development but instead for the global development of the adult head. This inference is based on the fact that the complete head loss of homozygous *eyg *mutants can be rescued by transient expression of *eyg *during both early as well as late eye antennal disc development [22]. In conclusion, the available data suggest that *eyg *has at least three separable patterning functions in the *Drosophila *eye-antennal imaginal disc, which concern the development of the entire head capsule, antenna and retina.

Here we present results from studying the postembryonic expression and function of the singleton *eyg *gene of the red flour beetle *Tribolium castaneum*. RNAi mediated lack-of-function phenotypes suggest that *Tribolium eyg *functions in the development of many structures including antenna, wing and eyes. Unexpectedly, *eyg *knockdown animals develop compound eyes with no evidence of growth impediment but surplus ommatidia instead. This phenotype is robust with regards to variation in dsRNA concentration, dsRNA fragment choice and injection time point. Testing further components of the N-dependent *Drosophila *eye growth network, we found that also the knockdown of *Jak *and *STAT*, individually or in combination, has no reducing effect on eye size in *Tribolium*. Morphogenetic analysis of the *eyg *knockdown eye enlargement phenotype revealed that the eye size increase occurs due to the lack of local inhibition of retinal differentiation by the developing gena. In summary, our findings provide compelling evidence that the gene regulatory network of adult eye growth control has substantially diverged between *Tribolium *and *Drosophila*, and reveal that *eyg *is an essential competence factor for the local suppression of retinal differentiation by the developing gena in *Tribolium*.

## Results

### Isolation and analysis of *Tribolium eyg *transcript sequences

**Figure 2:**
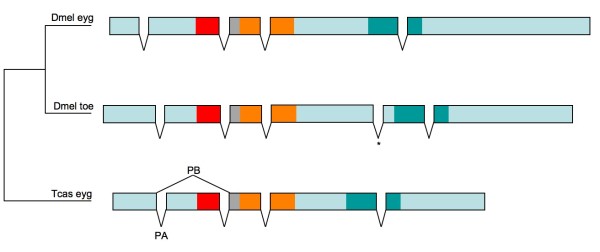
**Open reading frame organization in the *eyg *genes of *Drosophila *and *Tribolium***. The comparison between experimentally confirmed transcript sequences shows that all introns in the coding regions of *Drosophila *and *Tribolium eyg *homologs are conserved except for the acquisition of a new intron in the *Drosophila toe *gene (asterisk). Differential splicing in the 5' half of the *Tribolium eyg *transcript generates two predicted protein isoforms, one of which (Tc_*eyg*PA) contains a complete PD, whereas the other contains a truncated PD, which lacks the PAI subdomain (Tc_*eyg*PB). Protein domain regions are drawn to approximate relative scale and color coded: Red = PAI subdomain, grey = linker region, orange = RED subdomain, green = HD.

Previous bioinformatic searches identified candidate singleton orthologs of *eyg *in the genomes of mosquito, flour beetle, honeybee and the jewel wasp *Nasonia vitripennis *[26,27]. We performed RT-PCR and rapid amplification of cDNA ends (RACE) experiments on RNA isolated from embryos and pupal heads to test the NCBI *Tribolium eyg *gene model NM_001114345. RT-PCR and 3'-RACE products confirmed the predicted transcript sequence downstream of the RED subdomain. 5'RACE experiments recovered two classes of upstream cDNA sequences (Figure 2). One class (*Tc_eygPA*) corresponded to the *Tribolium eyg *NCBI gene NM_001114345 prediction, which includes open reading frame sequence that encompasses the complete PD region. The second class corresponded to a shorter transcript (*Tc_eygPB) *due to differential splicing of the conserved exon upstream of the PD-linker region. As a result, the protein isoform Tc_*eyg*PB is predicted to lack a sequence block immediately upstream of the linker-domain, which is conserved among insect *eyg *orthologs and has been speculated to possess a DNA-binding function [14], invoking the possibility that *Tribolium eyg *realizes diversified binding specificities of the PD by differential splicing.

### *Tribolium eyg *expression during pupal head development

**Figure 3:**
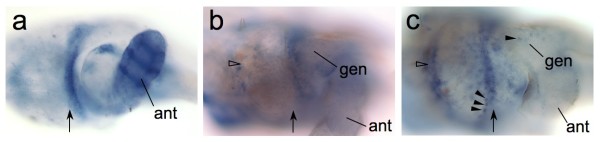
**Expression of *eyg *during adult head development in *Tribolium***. **(a-c) **Lateral view of larval or pupal *Tribolium *head labeled by whole mount *in situ *hybridization for expression of *eyg*. Arrows indicate anterior border of the differentiating adult eye primordium. Anterior is right and dorsal is up. **(a) **Late resting larva stage. Expression of *eyg *is detected in a dorsoventral domain between eye primordium and the antenna. **(b) **Early pupa at about 12 h after pupation. Open arrowhead indicates posterior edge of the differentiating retina. **(c) **Pupal stage about 24 h after pupation. Arrowheads point at small *eyg *expressing cell clusters at the anterior retina margin and in the epidermis of the gena. Ant = antenna, gen = gena.

The *Tribolium *compound eye develops in the lateral head epidermis of the late larva and the pupa [28]. To investigate whether *eyg *was expressed in this region, we performed whole mount *in situ *hybridization on larval and pupal head tissue preparations with a probe that extended from base pair 1 to 650 of the *Tribolium eyg *transcript NM_001114345. Earliest expression of *eyg *in the head epidermis was detected in the resting larva, which is characterized by the lack of food uptake and incipient metamorphosis (Figure 3a). Most prominent were expression domains in the developing antenna and in the area between the antenna and the presumptive eye primordium.

In the pupa, *eyg *expression appeared weaker and scattered more widely throughout the head epidermis (Figure 3b). The area between antenna and anterior eye margin continued to express *eyg*. In addition, small centers of localized expression could be detected in the epidermis posterior of the differentiating retina. After 48 hours of pupal development, elevated *eyg *expression was detected in a frame-like domain surrounding the entire differentiating retina. The number of small *eyg *expressing cell clusters seemed to have increased throughout the developing head epidermis (Figure 3c). A punctate pattern of *eyg *expressing cells or small cell groups was now also noted in the differentiating retina, suggestive of expression in ommatidial bristle cell precursors.

### Postembryonic knockdown of *eyg *results in a complex adult phenotype

**Figure 4:**
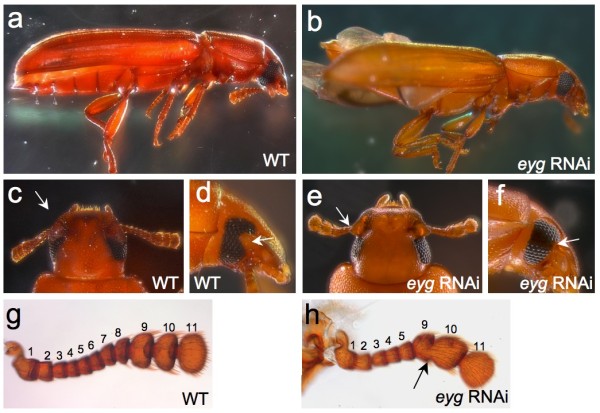
**The postembryonic *eyg *knockdown phenotype**. Extended focus depth stereomicroscope images of untreated and *eyg *knockdown *Tribolium*. **(a, b) **Whole body overview of untreated (a) and *eyg *knockdown (b) *Tribolium *from lateral perspective. **(c, d) **Head region of untreated *Tribolium *from (c) dorsal and (d) lateral perspective. **(e, f) **Head region of *eyg *knockdown *Tribolium *from (d) dorsal and (f) lateral perspective. Arrows point at gena. **(g, h) **Close up view of dissected antenna of (g) untreated and (h) *eyg *knockdown adult animal. Numbering of antennal segments based on [30]. Arrow points at segmental fusion.

To probe the requirement of *eyg *for the development of adult morphology in *Tribolium*, we induced systemic RNAi mediated gene knockdown in resting stage last instar larvae. The majority of adults hatching from injected larvae featured a combination of conspicuous morphological abnormalities (Figure 4). Most dramatically, *eyg *phenotypic animals were unable to fold their hindwings completely underneath the elytra in contrast to untreated animals (compare Figure 4a, b). Lateral inspection revealed that this defect was correlated with ventral protrusion of the second and third thoracic segment and the widening of the third thoracic segment (Figure 4b).

Three abnormalities characterized the head region of *eyg *phenotypic animals. From the dorsal perspective, it became apparent that the rim-like gena of untreated animals was deformed into a pointed, lateral extension (compare Figure 4c with 4e). From the lateral perspective, it could be seen that gena deformation was associated with the loss of the notched outline of the anterior adult eye in untreated animals (compare Figure 4d with 4f). In *eyg *phenotypic animals, the eye formed a linear anterior edge, apparently due to the failure of the gena to protrude into the anterior eye midline area as in untreated animals.

Consistent with the patterned expression of *eyg *in the developing pupal antenna (Figure 3a), we also noted defects in the antennae of *eyg *phenotypic adults, which included the reduction of proximal segments and partial fusion of terminal segments (compare Figure 4g with 4h). Previous authors recognized 11 segments in the *Tribolium *antenna [29,30]. The most proximal segments, scape and pedicel, were not affected in *eyg *phenotypic animals. The number of the ensuing funicle segments, however, was reduced from six down to three segments (Figure 4h). The three distal club segments were preserved in *eyg *phenotypic animals, but characterized by missing intersegmental articulations, frequently amounting to partial fusion of segments (arrow in Figure 4h).

### Postembryonic knockdown of *eyg *increases adult eye size

**Figure 5:**
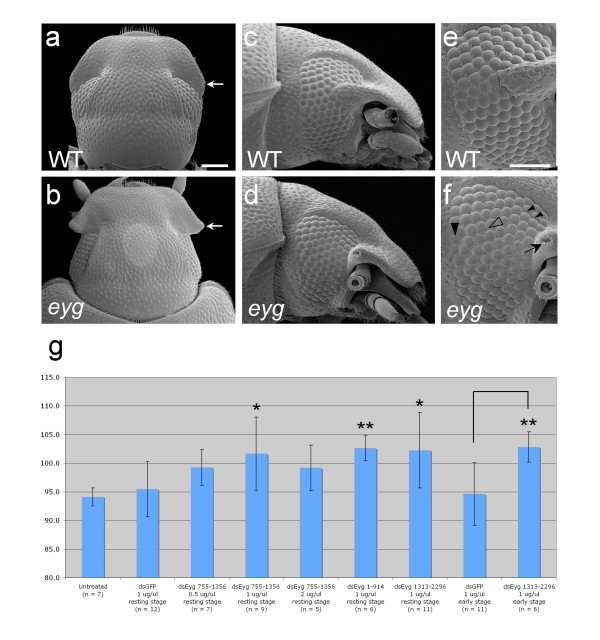
**Effect of postembryonic *eyg *knockdown on adult eye morphology in *Tribolium***. **(a-f) **Scanning electron microscopy images of adult head morphology. Scale bars corresponds to 100 μm. (a and b) Dorsal view of wild type (a) and *eyg *knockdown head (b). (c and d) Lateral view of wild type (c) and *eyg *knockdown head (d). (e and f) High magnification view of compound eye in wild type (e) and *eyg *knockdown (f) specimen. (e) Small arrowheads point at interommatidial bristles in the anterior dorsal and ventral regions of the compound eye where no bristles are formed in wild type animals. Large arrowhead points at irregularly shaped facet. Open arrowhead indicates multiplied interommatidial bristles. Arrow points at indenture at the tip of the extended gena. **(g) **Bar graphs showing comparison of compound eye size measured in average number of ommatidia of untreated and experimental animals injected with different dsRNA preparations and at different time points as indicated. Error bars represent standard deviation. Unlinked single asterisks indicate significant difference to EGFP dsRNA injected control based on two-tailed t-test (P < 0.05). Unlinked double asterisk indicates highly significant difference to untreated control based on two-tailed t-test (P < 0.005). Linked double asterisk indicates highly significant difference between EGFP dsRNA injected control and *eyg *dsRNA injected animals injected during early larval development based on two-tailed t-test (*P *< 0.005).

The apparent lack of regressive abnormalities in the compound eyes of *eyg *phenotypic animals was surprising given the strong *eyg*-dependence of eye primordium growth in *Drosophila*. We, therefore, investigated the morphology of the compound eye in *eyg *phenotypic animals in detail. Scanning electron microscopy confirmed the unusual lateral extension of the gena and the straightened anterior edge of the compound eyes (compare Figure 5a, c with 5b, d). High-resolution images further revealed that the facet surface of *eyg *knockdown animals was mildly roughened compared to untreated animals due to the occasional presence of ommatidia with irregularly shaped facet outlines (compare Figure 5c with 5d and 5e with 5f). A further abnormality could be noted regarding the distribution and organization of interommatidial bristles. In the posterior half of the compound eye of untreated *Tribolium*, a single interommatidial bristle is formed at each intersection point of four ommatidia. No interommatidial bristles, however, are present in anterior dorsal and ventral extensions of the eye field (Figure 5e). In *eyg *knockdown animals, interommatidial bristles were present across the entire retina (Figure 5f). Moreover, in some instances multiple interommatidial bristles were present at a single intersection point of four ommatidia (Figure 5f).

The interommatidial bristle phenotype was consistent with the expression of *eyg *in cell groups of the late differentiating retina detected by *in situ *hybridization (Figure 3c). This finding further implied that the developing retina cells experienced reduction of *eyg *transcript levels in experimental animals, which, however, did not result in expected growth-limiting effects. To the contrary, quantitative analysis of eye size measured by the number of ommatidia revealed on average a more than 5% higher number of ommatidia in phenotypic *eyg *knockdown animals (101.7 +/- 6.4) compared to untreated animals (94.1 +/- 1.6) or animals control-injected with Enhanced Green Fluorescent Protein (EGFP) dsRNA (95.5 +/- 4.8) (Figure 5g), consistent with the anterior extension of the retina in *eyg *knockdown animals.

Asking whether the effect of *eyg *reduction by systemic RNAi knockdown was dosage dependent, we performed knockdown experiments with dsRNA concentrations ranging from 0.5 μg/μl to 2 μg/μl dsRNA. The same eye and antennal abnormalities were obtained with each concentration, further corroborating the conclusion that the developing *Tribolium *eye was insensitive to *eyg *reduction (Figure 5g). Injecting larvae with dsRNAs targeting additional non-overlapping regions of the *Tribolium eyg *transcript generated similarly significant average enlargement of the eye (Figure 5g), ruling out indirectly eye-size rescuing effects by off-target down-regulation of modifier loci.

Finally, we probed whether a possible eye-growth promoting requirement of *eyg *occurred at earlier time points of postembryonic development by comparing the effect of injecting larvae smaller than 3.2 mm body length with EGFP control dsRNA and *eyg *dsRNA. Eye morphologies and average eye sizes obtained from *eyg *dsRNA injected larvae in these experiments were not significantly different from late injected larvae but significantly larger compared to the control injected animals (Figure 5g). Taken together, these results strongly suggested that *eyg *was dispensable for postembryonic eye primordium growth in *Tribolium *in contrast *Drosophila*.

### Postembryonic knockdown of *Jak *and *STAT *has no effect on eye development

**Figure 6:**
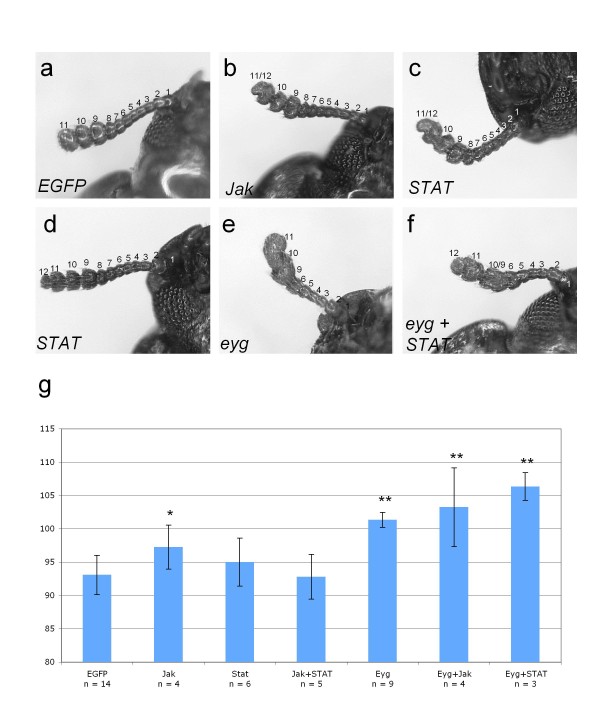
**Effect of postembryonic knockdown of *Jak *and *STAT *on adult antenna and eye development**. **(a-f) **Antenna of adult *Tribolium*. **(**a) Antenna of EGFP dsRNA control injected animal. (b) Antenna of *Jak *knockdown animal with a partially subdivided or duplicated terminal segment. (c) Antenna of *STAT *knockdown phenotypic animal with a partially subdivided or duplicated terminal segment. (d) Antenna of *STAT *knockdown phenotypic animal with a fully subdivided or duplicated terminal segment. (e) Antenna of *eyg *knockdown animal with reduced number of funicle segments and partially fused club segments (compare with Figure 4h). (f) Antenna of *eyg+STAT *knockdown animal showing the combination of *STAT *and *eyg *knockdown-induced antenna defects. Similar combinatorial phenotypes were observed in *eyg+Jak *knockdown animals. **(g) **Bar graphs showing comparison of compound eye size measured in average number of ommatidia between untreated and experimental animals injected with different dsRNA preparations as indicated at 1 μg/μl concentration. Error bars represent standard deviation. Single asterisks indicate significant difference to EGFP dsRNA injected control based on two-tailed t-test (P < 0.05). Double asterisks indicate highly significant difference to EGFP dsRNA injected control based on two-tailed t-test (P < 0.005).

In *Drosophila*, the eye disc growth promoting effect of *eyg *is induced via the induction of *upd *transcription and the activation of *Jak/STAT *signaling [7]. To further explore whether the *eyg*-related genetic regulation of the adult eye growth differed between *Drosophila *and *Tribolium*, we isolated cDNA fragments of the *Tribolium *orthologs of *Jak *and *STAT *and probed their requirement during postembryonic development by systemic RNAi mediated knockdown. Single knockdown of *Jak *or *STAT *as well as double knockdown of both genes did not result in conspicuous morphological abnormalities. Detailed examination, however, revealed effects in the distal antenna, which were distinct from *eyg *knockdown animals. In both *Jak *and STAT phenotypic animals, the distal-most club segment appeared to be partially or completely subdivided or duplicated, generating the impression of an additional segment (Figure 6a-d). This abnormality was observed in both *Jak *and *STAT *knockdown animals consistent with the operation of both genes in the same signaling pathway. The penetrance of this antenna phenotype was low, ranging around 25% in *Jak*, *STAT *or *Jak+STAT *double knockdown animals (Table 1). Using the antenna phenotype as an internal marker of *Jak *and *STAT *knockdown efficiency, we compared average eye size of *Jak*, *STAT *or *Jak+STAT *phenotypic animals to control injected animals. Average eye size in any of these experimental treatments was not significantly reduced compared to control animals (Figure 6g). Eye size was marginally significantly increased in *Jak *knockdown animals; however, this was not reproduced in *Jak/STAT *knockdown experiments. These results suggested that, like *eyg*, *Jak *and *STAT *were not essential for eye growth in *Tribolium*.

**Table 1:** Lethality and antenna phenotype penetrance in *eyg*, *Jak *and *STAT *interaction knockdown analysis.

	*EGFP*	*eyg*	*STAT*	*Jak*	*Jak/STAT*	*eyg/Jak*	*eyg/STAT*
Animals injected	23	20	57	60	58	56	55
Lethality	39%	40%	56%	68%	67%	41%	65%
Penetrance of *eyg *antenna knockdown phenotype	0	75%	0	0	0	24%^1^	32%^1^
Penetrance of *Jak *and *STAT *knockdown antenna phenotype	0	0	24%	21%	26%	24%^1^	21%^1^
Penetrance of *eyg/Jak/STAT *knockdown antenna phenotype	0	0	0	0	0	12%	16%

Data derived from knockdown experiments described in Figure 6g.

^1 ^Includes animals exhibiting specific or combinatorial phenotype

### Combinatorial knockdown of *eyg *with *Jak *or *STAT*

To test the possibility that *eyg *operated in parallel and redundantly with *Jak/STAT *signaling in *Tribolium *eye development, we examined the effect of knocking down *eyg *in combination with *Jak *and *STAT*. Using the antennal phenotypes as markers of single and combinatorial knockdown animals (Figure 6b-f), we compared average eye size between phenotypic *eyg *single knockdown animals and double knockdown animals. All three treatments resulted in a significant increase of average eye size compared to control-injected animals, while no significant difference in eye size was observed between *eyg *single knockdown animals and *eyg+Jak *or *eyg+STAT *animals (Figure 6g). These results suggested that *eyg *did not interact with *Jak/STAT *signaling in the *Tribolium *eye and that both genetic components are not involved in the activation of primordium growth during *Tribolium *eye development.

Interestingly, the penetrance of *eyg *knockdown induced antennal reduction was conspicuously higher in single knockdown animals (75%) than in the two combinatorial knockdown treatments (<35%) (Table 1). This indicated an antagonistic interaction between *eyg *and *Jak/STAT *signaling during antenna development, consistent with the opposite effects of the respective knockdown treatments on the development of antennal segment number (Figure 6b-f).

### Postembryonic knockdown of *eyg *prevents localized repression of retinal differentiation

**Figure 7:**
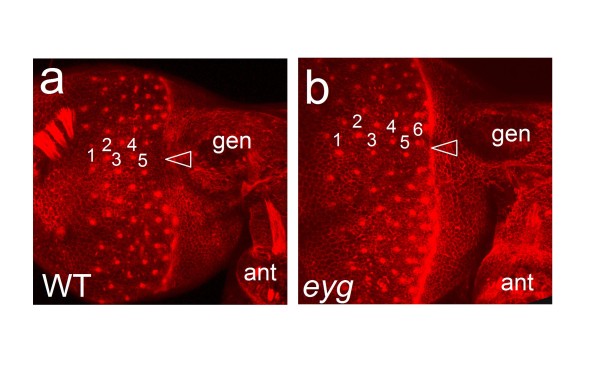
**Eye morphogenesis in *Tribolium eyg *knockdown pupae**. **(a, b) **Confocal images of lateral head of phalloidin labeled 24 h old *Tribolium *pupa of wild type (a) and *eyg *knockdown (b) specimens. Numbers indicate vertical columns of differentiating ommatidial precursor clusters. Arrowheads point at midline area of the anterior differentiating retina. ant = antenna, gen = gena.

The development of the *Tribolium *retina begins with the anterior progression of a differentiation front in the posterior head epidermis similar to the morphogenetic furrow in the *Drosophila *eye disc, which can be visualized by phalloidin staining of filamentous actin [31]. Also similar to *Drosophila*, the retinal differentiation front initiates as a continuous dorsoventral line in *Tribolium*. Unlike in *Drosophila*, however, the retinal differentiation front of *Tribolium *separates into independent dorsal and ventral fronts by the time the first five rows of ommatidial preclusters have been formed (Figure 7a) [31]. The split of the *Tribolium *retinal differentiation front occurs in the midline region of the anterior eye and correlates spatially with the protrusion of the expanding gena into the retina.

Noting the correspondence between the area taken up by the gena in the anterior eye of wild type *Tribolium *and the location of additional ommatidia in *eyg *knockdown phenotypic animals, we speculated that the eye expansion phenotype resulted either from ectopic induction of ommatidia or from uninterrupted retinal differentiation in this area. To probe for these scenarios, we examined the cellular morphogenesis of the differentiating retina in phenotypic *eyg *knockdown pupa by phalloidin staining. In contrast to the subdivided morphogenetic furrow in untreated pupae, the morphogenetic furrow remained contiguous in *eyg *knockdown pupae even after the establishment of more than five ommatidial precluster rows (compare Figure 7a, b). This finding supported the model that the eye size increase in *eyg *knockdown animals resulted from continued retinal differentiation in the anterior midline area.

## Discussion

### *Eyg *is a pleiotropic regulator in insect adult body plan development with shared and diverged roles in *Tribolium *and *Drosophila*

Our postembryonic analysis of *eyg *function in *Tribolium *revealed diverse *eyg*-dependent adult patterning events including, and presumably not limited to, the development of antennae, hind wings, gena and the ventral thorax. In *Drosophila*, *eyg *and its closely related paralog *toe*, have also been found to be associated with a diversity of patterning processes. Interestingly, however, the findings in this study and the available data from *Drosophila *suggest only a limited overlap between the two species. The most likely example of functional conservation concerns the role of *eyg *in the developing antenna. In *Drosophila*, *eyg *requirement has been reported for both the specialized larval antennal organs and the adult antenna [9,32]. In preliminary experiments, we also find evidence for *eyg *requirement in the larval antenna of *Tribolium *(XY and MF, unpublished). The dramatic modification of the aristate adult antenna of *Drosophila *in contrast to the ancestrally organized capitate adult antenna of *Tribolium*, however, prevents straightforward inferences about the specific nature of the conserved functions of *eyg *in antennal development. Future studies may unravel specific *eyg*-dependent patterning processes in the developing antenna of both species. Alternatively, *eyg *may function as a competence factor in both systems. The differential expression of *eyg *in the developing *Tribolium *antenna, however, speaks against this scenario.

Other effects of *eyg *knockdown on adult body plan patterning in *Tribolium *show less correspondence to *Drosophila*. Mutant *Drosophila *homozygous for weak *eyg *alleles are characterized by partial or complete absence of the compound eyes [17]. Strong *eyg *alleles are associated with the complete absence of eye-antennal imaginal disc derived head structures, defective salivary gland development, and notum mispatterning in the mesothoracic segment [17,32,33]. We were not able to investigate the postembryonic development of salivary glands in *Tribolium *leaving it to further investigation to discover if *eyg *performs conserved functions in this context. However, no patterning defects could be detected in the dorsal thorax of *Tribolium*, suggesting a regulatory difference between the two species.

The insensitivity of eye primordium growth in *Tribolium *marked a second major difference to *Drosophila*. The same can be stated for the lack of a detectable role of *Tribolium eyg *in global head development. However, our experiments did not comprehensively address the question of whether *eyg *functions in *Tribolium *head development. In *Drosophila*, this function is genetically separable from *eyg*'s role in eye primordium growth [22]. Short pulses of *eyg *expression throughout embryonic and postembryonic development are sufficient to rescue head development from the eye antennal disc in *Drosophila *[22]. The lack of effects on head development in our experiments could, therefore, be due to the enduring effect of *eyg *expression before the initiation of knockdown. Second, in contrast to *Drosophila*, all basic components of the head capsule become fully developed during embryogenesis in *Tribolium *[34]. Consistent with this, previous work showed that *Tribolium *head development is sensitive to the downregulation of *ey *and *toy *in the embryo but not in the pupa, while the consequence of *ey *or *toy *reduction on head development becomes manifest during postembryonic development in *Drosophila *[35]. The contribution of *eyg *to global head patterning in *Tribolium*, therefore, needs to be further investigated by embryonic knockdown analysis. Preliminary results of experiments in this direction are not indicative of a role of *eyg *in the global patterning of the *Tribolium *larval head (XY and MF, unpublished).

### The role of *Drosophila eyg *and *Jak/STAT *signaling in promoting eye primordium growth is not conserved in *Tribolium*

The primary motivation of our study was the question of whether *eyg *is an important facilitator of adult eye primordium development in *Tribolium *like in *Drosophila*. Surprisingly, our results suggest that this is not the case as phenopypic *eyg *knockdown *Tribolium *are characterized by eye gain instead of loss. In *Drosophila*, the development of the adult eye is not affected in heterozygous *eyg *mutants [8,9], indicating considerable tolerance of the eye-antennal disc related patterning processes to *eyg *reduction. Genetic interaction analysis, however, has shown that the developmental robustness of *eyg *heterozygous animals is at least partly due to compensatory regulation by *toe *[9]. Moreover, eye size reduction manifests itself already in weak *eyg *alleles in *Drosophila*, while the complete head loss phenotype is only seen in null mutant *eyg *lines [17]. These data suggest that the eye proliferation growth is less tolerant to *eyg *activity reduction than other *eyg *regulated processes. Nonetheless, the possibility needs to be considered that the lack of eye reduction effects in *Tribolium eyg *knockdown animals may be due to insufficient reduction of target transcript levels by the RNAi treatment. Several lines of evidence suggest that this is unlikely: (I) The complex phenotype of *eyg *knockdown in *Tribolium *affecting multiple organs and structures speaks to the general sufficiency of the mediated transcript reduction by systemic RNAi for perturbing *eyg *dependent processes. (II) The interommatidial bristle abnormalities discount the possibility of local knockdown insensitivity in the retina. (III) Consistent with this, previous experiments targeting members of the retinal determination gene network demonstrated high sensitivity of *Tribolium *adult eye development to RNAi mediated down-regulation of eye developmental genes [35,36]. (IV) Although we observed variation in the significance of average eye size increase most likely due to sample size variations (Figure 5g and 6g), the penetrance and expressivity of the *eyg *knockdown phenotype was consistent over a range of injected dsRNA concentrations as well as injection time points. (V) Also the knockdown of *Jak *or *STAT*, which are downstream facilitators of *eyg *dependent growth in the *Drosophila *eye disc, had no detectable consequences on adult eye development in *Tribolium*. (VI) This was even the case when *Jak *was co-targeted with *STAT *or *eyg*, further reducing the possibility that the lack of phenotype may be due to low transcript threshold requirements of eye-related functions of these genes.

It is conceivable that more regressive eye defects are hidden in larval or pupal lethal knockdown phenotypes. However, compared to the 40% lethality in control-injected animals the lethality in *Jak *or *STAT *dsRNA injected animals was increased by less than 20% and no difference was observed in *eyg *single knockdown animals (Table 1). Moreover, we did not observe developing knockdown pupae with overt retinal patterning defects, which can be conveniently monitored in *Tribolium *by peripheral inspection [36]. While the results of our knockdown experiments can not entirely rule out a role of *eyg *or *STAT *and *Jak *in *Tribolium *eye primordium growth that is robust to strong transcript reduction, it may be noted that the results of RNAi mediated lack of function analysis has been considered sufficient evidence for a lack of *toe *involvement in *Drosophila *eye development [9]. We, therefore, conclude that our results provide compelling evidence that neither *eyg *nor *Jak/STAT *signaling is involved in growth activation during *Tribolium *adult eye development.

### Evolutionary scenarios explaining the divergence of postembryonic eye primordium growth control in *Drosophila *and *Tribolium*

The contrasting roles of *eyg *with regards to the postembryonic growth of the adult eye primordium in *Drosophila *and *Tribolium *generate the question: Which state is ancestral? Two scenarios need to be considered, given the particularities of the eye developmental process in these species. In *Drosophila *and other cyclorrhaphan Diptera, the development of the adult eye requires rapid and massive cell proliferation in the eye-antennal imaginal disc [8]. The ancestral state of postembryonic adult eye development in endopteryote species, however, is represented by the placode-like primordium in the peripheral larval head cuticle of species like *Tribolium *[34]. *Drosophila*-like internalized imaginal disc tissues have multiple times evolved in other endopterygote insect groups, but independently in parallel [37]. It is, therefore, conceivable that the involvement of *eyg *and Jak/STAT signaling originated specifically during dipteran eye-antennal imaginal disc evolution. In this case, the lack of *eyg *and *Jak/STAT *signaling involvement in the *Tribolium *eye would represent an ancestral state. Further evidence in support of this may be seen in the fact that the expression patterns of all orthologs of *Drosophila *N-dependent eye growth network genes so far examined in the directly developing grasshopper species *Schistocerca americana *are inconsistent with functional conservation of this gene regulatory network in the embryonic retina primordium of this species [38]. However, while these data speak against a deeper evolutionary conservation of N-dependent eye growth, it must be kept in mind that the embryonic phase of eye development in directly developing insects is not orthologous to the postembryonic phase of eye development in endopterygote insects both with respect to timing as well as the mechanisms of retinal precursor tissue formation [34].

There is, therefore, a second possibility that needs to be considered: The ancestral mode of postembryonic eye development in endopterygote insects may be represented by a peripheral adult eye primordium, which proceeds through a transient stage of disc-like tissue invagination to accommodate for the massive tissue expansion within the confinement of the larval head capsule {Truman, 2002 #5919}. This type of postembryonic eye morphogenesis occurs in endopterygote species with large adult eyes, such as the tobacco hornworm *Manduca sexta *[39]. Counting on average less than 100 ommatidia, the compound eyes of *Tribolium *are exceptionally small. For comparison, the compound eyes of *Drosophila *and *Manduca *consist of over 800 and 25,000 ommatidia, respectively [40]. Consistent with these size differences, the developing eye primordium of *Tribolium *exhibits no indication of transient invagination [41]. Cell proliferation has been observed anterior and posterior of the morphogenetic furrow of *Tribolium*, suggesting some degree of postembryonic primordium growth [41]. Notwithstanding this, however, it is possible that the lack of *eyg *involvement in the eye primordium growth of the small-eyed *Tribolium *is derived, while the N-dependent genetic network involving *eyg *and *Jak/STAT *signaling is ancestrally required for the postembryonic growth of large retinal primordia in endopterygote insects. Additional comparative investigations in endopterygote insects will thus be required to clarify with certainty whether the growth-related functionality of *eyg *in the developing eye of *Drosophila *is derived or not.

### Eye versus gena development in *Tribolium*: a model

Our experiments opened unexpected insights into an as yet little studied aspect of *Tribolium *adult head development, which involves the characteristically notched outline of the anterior eye and the posterior protrusion of the gena. The comparison of retinal development in normal and *eyg *knockdown *Tribolium *revealed that the introgression of the gena involves the local termination of retinal differentiation in the anterior midline of the developing eye, the contact zone between gena and retina. One possible explanation of the enlargement of the retina in conjunction with the malformation of the gena is that *eyg *is required for normal development of the latter. Our expression analysis, however, revealed that *eyg *is not expressed in the gena itself. This suggests that *eyg *does not directly promote the specification, growth or morphogenesis of the gena. Consistent with this, the gena of *eyg *knockdown animals appears strongly deformed but not overtly reduced, as would be expected from autonomously or non-autonomously induced defects in the regulation of growth or regional specification.

**Figure 8:**
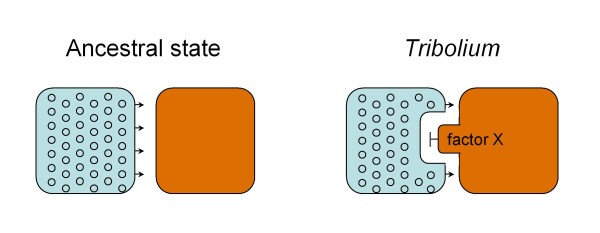
**A model of gena development and evolution in *Tribolium***. In the ancestral state, retinal differentiation progresses at equal rate and distance along the anterior edge of the developing eye. In *Tribolium*, retinal differentiation is suppressed in the anterior midline area of the developing eye by an unknown signal (factor X) produced in the posteriorly expanding gena. The results of this study further show that *eyg *is essential for the competence of cells in the contact zone between retina (blue field) and gena (brown field) to respond to factor X.

At the time point of early retinal development, *Tribolium eyg *is expressed in a dorsoventral domain that straddles the entire anterior margin of the developing eye. This expression domain includes, but also exceeds, the contact zone of gena and retina dorsally and ventrally. This implies that *eyg *is not involved in promoting retinal differentiation, because in this case retinal differentiation would be expected to abort prematurely along the entire retinal progression front in *eyg *knockdown animals. The relationship between the spatial distribution of *eyg *transcript and the affected structures in *eyg *knockdown animals instead supports a model in which *eyg *provides cells anterior of the retinal differentiation front with the competence to respond to a retina-differentiation suppressing signal that is released from the expanding gena (Figure 8). In the absence of *eyg*, the tissue between retina and gena is 'blind' to this signal and retinal differentiation is allowed to progress through the midline region. This model and the anterior compression of the gena in *eyg *knockdown animals further imply that retina development is epistatic of gena development in the absence of *eyg*.

Interestingly, the default priority of retina over gena fate is expected from a comparative evolutionary perspective. Since the posteriorly extended gena of *Tribolium *and related darkling beetles is a derived trait (Figure 8), its development must have been superimposed onto the more ancestral gene regulatory module promoting retinal development during evolution. However, while the proposed model explains the experimental data and their evolutionary developmental basis, questions remain. One is why the evolution of gena protrusion into the retina involved the implementation of a competence mechanism while the required block of retinal differentiation could be more parsimoniously implemented through direct suppression by a signal from the gena. Another question concerns the possible functions of *eyg *expression in front of the differentiating retina outside the gena-retina contact area. One possibility is that *eyg *may affect early retinal patterning steps, possibly reflected by the facet and bristle irregularities in the retina of *eyg *knockdown animals.

The molecular nature of the signal, which is produced by the gena to delimit retinal differentiation (factor X in Figure 8), is left to speculation at this point. Published expression patterns of *wg*, a conserved candidate antagonist of retinal differentiation in *Tribolium*, do not support a role of this signaling factor in the developing gena [42]. It is tempting to hypothesize a role of *homothorax *(*hth*) in the local repression of retina differentiation given its antagonistic role in the *Drosophila *eye developmental gene network [43]. Future efforts in elucidating the antagonistic interplay between gena and retina development during eye notch formation in *Tribolium *would open the door to comparative studies of the origin of this morphological novelty in related darkling beetles. At this point, it is tempting to speculate that the evolution of the extended *Tribolium *gena was driven by functional advantages related to the digging behavior of this species but possibly also by energy savings associated with reduction of the visual system [44]. The latter is expected to play a role in the adaptive evolution of opportunistic detritivorous colonizer species like *Tribolium *[45].

## Conclusions

The *Tribolium *singleton ortholog of *eyg *is essential for normal development of the hind wings, thorax, antennae and the eye. In contrast to *Drosophila*, *eyg *plays a delimiting role in *Tribolium *eye development, facilitating the local repression of retinal differentiation associated with the posterior extension of the gena. While further comparative data will be needed to clarify the evolutionary ancestry of the N-dependent adult eye primordium growth gene network module in the *Drosophila *eye disc, these findings make a first dent in understanding the developmental evolution of the characteristic *Tribolium *eye notch, thereby identifying a new opportunity for studying the developmental evolution of a novel trait in this resourceful model. Comparative analyses within the highly diverse darkling beetles would 'root' the *Tribolium *state and prepare the entry point for functional genetic studies of adaptive trends and causes in the evolution of eye size and shape in this group.

## Methods

### Animal strains and culture

Cloning experiments were carried out with animals from a colony of *Tribolium castaneum *obtained through Carolina Biological Supplies (Burlington, North Carolina, USA). In all other experiments we used the Georgia 1 (GA 1) or *pearl *pBac(3xP3-EGFP)af strains. All stages of the *Tribolium *cultures were maintained in constant darkness on yeast enriched whole-wheat flour as described previously [31].

### Molecular biology

Expression of *Tribolium eyg *was investigated by RT-PCR with upstream primer GTGTGTGCACTAATGCGACG and downstream primer AGTAGTAAGCGGCGAGCCAG, which amplify a 651 base pair long region that spans from the RED subdomain to the HD. Total RNA from *Tribolium *embryos and pupae was extracted with the RNAqueous kit (Applied Biosystems/Ambion, Austin, Texas, USA) and used for random decamer primed cDNA synthesis with the RETROscript kit (Ambion). A total of 1 μl cDNA product was used as a template for PCR amplification, which was carried out with the following cycle conditions: denaturation for 60 seconds at 94°C, annealing for 60 seconds at 45°C to 55°C, elongation was held for 60 seconds. PCR amplicons were examined by gel electrophoresis and cloned into pGEM-T vector (Promega, Fitchburg, Wisconsin, USA). Eight clones from two independent PCR reactions were sequenced with the BigDye Terminator sequencing kit (Applied BioSystems, Foster City, California, USA) and forwarded for electrophoretic separation to the Applied Genomics Technology Center of Wayne State University. Further transcript sequence was isolated through 5' and 3'RACE experiments using the FirstChoice RLM-RACE kit (Ambion). Oligonucleotides used in 3'RACE were GTTCGAGAAGAGCCACTATC and AGGCAGCCAGTCTTCGATGTT. Oligonucleotide combinations used in 5'RACE were GCCAGGAGGTGAGTTGAGAA and GTTAGGCCACAGAGGTGAAT; CGGAAAGTCCAGGAACATTG and GCGTTTCCGTTGGAGATTAG; GCCGTAGAGAGAACACTGG and CGGAAAGTCCAGGAACATTG; GGTGGTAGAGGCCATAGC and GAGCCGTCGCATTAGTGC. Sequence comparison, contig assembly and open reading frame analysis were performed in MacVector 6.0.1 (MacVector Incorporated, Cary, North Carolina, USA) and submitted to GenBank (EU186803, EU186804, EU186802). Sequence fragments of the *Tribolium *orthologs of *Jak *and *STAT *were cloned by RT-PCR as described above using the primer combination GTCCAGTAATCGCAGCCAAT and AGTAGTAAGCGGCGAGCCAG for *Jak*, and GAAAAACAACCGCCACAAGT and GACTGAGAAGGGCACTCGAC for *STAT*. The cloned cDNA fragments of both genes were sequenced and found identical with the NCBI mRNA sequence predictions for *Tribolium Jak *(EFA07411) and *STAT *(XM_964384).

### *In situ *hybridization

Whole mount *in situ *hybridization experiments on *Tribolium *postembryonic head tissues were carried out following a previously published protocol [42]. Digoxigenin labeled RNA probe of the *Tribolium eyg *transcript was synthesized by *in vitro *transcription using standard reagents from Roche Applied Science (Indianapolis, Indiana, USA).

### Knockdown analysis

Larval RNAi experiments were carried out following published protocols [46]. dsRNA was prepared by *in vitro *transcription with T7 RNA polymerase (Ambion) from PCR generated template DNA that was flanked by T7 site-carrying amplification primer sequence. Three different dsRNA preparations were produced corresponding to nucleotides 1-914, 755-1356 and 1313-2296 of *Tribolium eyg *NCBI transcript sequence NM_001114345. The dsRNA *STAT *and *Jak *preparations encompassed the regions of the respective cloned cDNA fragments, which were 498 and 449 bp long, respectively. Injections were delivered at 1 μg/μl dsRNA concentration unless specified otherwise.

### Phalloidin staining

Whole mount labeling of postembryonic *Tribolium *head tissue was carried out as previously described [42]. The heads of last instar *Tribolium *larvae and early pupae were fixed for 30 minutes in 3.7% formaldehyde in 1 × PEMS buffer (100 mM PIPES, pH 6.9, 1 mM EDTA, 1 mM MgCl2, and 1.2 M sorbitol) at room temperature. Fixed tissues were washed three times in PBT followed by overnight incubation at 4°C in PBT containing rhodamin-conjugated phalloidin (Molecular Probes, Eugene, Oregon, USA) at 1:30 dilution.

### Brightfield microscopy

The *in situ *hybridization labeled tissues were studied on a Zeiss Axioscope (Jena, Germany) with differential interference optics. Digital images were taken with a SPOT RT color camera (Diagnostic Instrumental Incorporated, Sterling Heights, Michigan, USA). Stereomicroscope image stacks were taken with a Leica DFC490 camera coupled to a Leica M216 A stereomicroscope (Leica Microsystems, Wetzlar, Germany). Post-imaging extension of focal depth was carried out using the Montage module integrated in the Leica Application Suite package. Adjustments of brightness and contrast were carried out in Photoshop CS3 (Adobe Systems Incorporated, San Jose, California, USA).

### Laser scanning confocal microscopy

Phalloidin labeled specimens were cleared in 70% glycerol in PBS supplemented with 10% DABCO and mounted with peripheral cover slip support by an applied ring of Vaseline^®^. Stacks of confocal images were taken on a Leica TCS SP2 laser scanning confocal microscope. Image projections were generated using the Leica Confocal Software package (Leica Microsystems, Wetzlar, Germany).

### Scanning electron microscopy

Adult *Tribolium *head cases were cleaned with ultrasonic sound, dehydrated in an ethanol series, dried, and coated with gold (EmiTech K500 sputter coater) (EmiTech, Ashford, Kent, United Kingdom). Images were taken on a Philips XL 30 ESEM and analyzed using the Scandium 5 software package (Amsterdam, Netherlands).

### Quantitative analysis of adult eye size

Adult eye size was measured by numbers of ommatidia as previously described [35]. Individual counts were replicated by different individuals.

## Abbreviations

EGFP: Enhanced Green Fluorescent Protein; eyg: eyegone; ey: eyeless; HD: homeodomain; hth: homothorax; Jak/STAT: Janus kinase/Signal Transducer and Activators of Transcription; N: Notch; PD: paired domain; RACE: rapid amplification of cDNA ends; sv: shaven; upd: unpaired; toe: twin of eyeless; toy: twin of eyeless; wg: wingless;

## Competing interests

The authors declare that they have no competing interests.

## Authors' contributions

XY cloned the *Tribolium eyg *cDNAs, performed the expression analysis and investigated the *Tribolium eyg *knockdown phenotype. NZ investigated dosage and time point dependence of *eyg *kockdown, cloned the cDNAs of *Tribolium Jak *and *STAT *and performed the knockdown analysis of the latter two genes in combination with *eyg*. FF and RB generated the electron microscopy images. RB added 5'RACE sequences and analyzed the genomic organization of the *Tribolium eyg *coding sequence. MF conceived the project and wrote the manuscript.
